# Outcome in Cats with Acute Onset of Severe Thoracolumbar Spinal Cord Injury Following Physical Rehabilitation

**DOI:** 10.3390/vetsci8020022

**Published:** 2021-01-29

**Authors:** Antonella Gallucci, Ludovica Dragone, Tania Al Kafaji, Marika Menchetti, Sara Del Magno, Gualtiero Gandini

**Affiliations:** 1 Neurology Service, La Fenice Veterinary Center, 09032 Assemini, Italy; 2 Physiotherapy and Rehabilitation Center “Dog Fitness,” 42124 Reggio Emilia, Italy; info@dogfitness.it; 3 Department of Veterinary Medical Sciences, University of Bologna, 40064 Ozzano Emilia, Italy; tania.alkafaji@hotmail.com (T.A.K.); sara.delmagno@unibo.it (S.D.M.); gualtiero.gandini@unibo.it (G.G.); 4 Neurology and Neurosurgery Division, San Marco Veterinary Clinic, 35030 Veggiano, Italy; menchettimarika@gmail.com

**Keywords:** absent deep pain perception, acute onset of thoracolumbar injury, paraplegic cats, spinal walking

## Abstract

The literature is lacking data concerning the prognosis in cats suffering from naturally occurring acute onset of thoracolumbar (TL) spinal cord injury that are undergoing rehabilitation therapy. Therefore, we investigated the effect of physical rehabilitation in cats suffering from naturally occurring TL spinal cord injury. The medical records of 36 cats with acute onset of TL spinal cord injury that were selected for rehabilitation treatment were reviewed. Twenty-nine cats underwent an intensive physical rehabilitation protocol in the clinic (group 1), whereas the owners of seven cats declined physical rehabilitation (group 2). In group 1, seven cats had pelvic limb deep pain perception (DPP), which was significantly associated with the functional recovery of voluntary ambulatory status (*p* = 0.010) and voluntary micturition (*p* < 0.001). Spinal walking was achieved in 10/22 (45%) of the cats without DPP, and none regained voluntary micturition. In group 2, no cats regained ambulatory status or voluntary micturition, although pelvic limb DPP was present in three patients. Treatment with a clinic-based rehabilitation program and the presence of a crossed extensor reflex were significantly associated with a higher possibility of regaining functional ambulatory status (*p* < 0.010), but there was no difference in the recovery of voluntary micturition between the groups. Thus, cats with severe, naturally occurring, acute onset of TL spinal cord injury may benefit from physical rehabilitation. In the case of the loss of DPP, the acquisition of spinal walking is possible, despite the high possibility of a persistent neurologically dysfunctional bladder.

## 1. Introduction

In the last decade, the use of physical therapy in veterinary medicine has increased and an abundance of research describing its role in the outcome of dogs with thoracolumbar (TL) intervertebral disk herniation (IVDH) has been published [1,2,3,4,5,6]. Physical therapy also seems to have a beneficial role in dogs with severe traumatic injuries of the spinal cord [5]. In cats, many studies performed under experimental conditions have shown the usefulness of treadmill activity in promoting the recovery of motor function after incomplete and complete spinal cord lesions [7,8,9].

Rehabilitation seems most beneficial in dogs with a functionally complete spinal cord injury, highlighting the possibility of achieving an involuntary reflex gait, commonly described as spinal walking (SW) [5]. In the absence of superior control by the brain after a complete spinal cord injury, the acquisition of SW is made possible by dynamic interactions between the pelvic limb central pattern generator and proprioceptive feedback from the body [10,11].

To the best of the authors’ knowledge, the veterinary literature is lacking data related to the role of rehabilitation in cats with naturally occurring, acute onset of TL spinal cord injury. Therefore, this study aimed to retrospectively investigate the results of physiotherapy in cats suffering from naturally occurring, acute onset of TL spinal cord injury and to evaluate their outcome in regard to deep pain perception (DPP) and performance of physiotherapy.

## 2. Methods

A retrospective study was conducted on feline patients with acute onset of TL spinal cord injury that were referred to the Physiotherapy and Rehabilitation Center “Dog Fitness” (Reggio Emilia, Italy) between 2006 and 2017. Cats were considered eligible for the study if they satisfied the following criteria: (1) the presence of complete clinical records, including physical examination data and neurological status, assessed by a neurological examination performed by the neurologist at admission and at the end of the treatment; (2) diagnosis of acute onset of TL spinal cord injury, confirmed by survey radiography of the spine (in the case of external trauma) or advanced imaging techniques (magnetic resonance imaging or computed tomography); (3) availability of data regarding the physical rehabilitation protocol performed.

Cats that met the criteria and followed clinic-based physical rehabilitation were designated as group 1, and cats that met the first two criteria but whose owners declined rehabilitation treatment because of financial issues were enrolled in the study as group 2. The latter was used as a control group. In group 2, the owners were instructed on how to manage urination, but they did not perform any exercises on the cats at home. Data regarding further improvement were collected through recorded follow-up neurological consultations for group 1. The information on group 2 was gathered by writing a record of the first examination at the time of the patient’s admission and by interviewing the owners by phone at the time of this study, with the aim of collecting data regarding the acquisition, or not, of an involuntary ambulatory status (outcome). Ambulatory status was defined as the ability to rise and take at least 10 consecutive weight-bearing steps unassisted without falling [12]. Cats without a minimum of a 3-month follow-up were excluded. In the case of the absence of recorded data, follow-up information was obtained by interviewing the referral neurologist at the time of this study.

The rehabilitation program was established and personalized by the physiotherapist for each patient and included the following basic categories of exercises: passive range-of-motion exercises, flexor reflex and crossed extensor reflex stimulation, active-assisted exercises, and hydrotherapy with an underwater treadmill. Overall, cats underwent 45–60 min of physical rehabilitation treatment, twice daily, 7 days per week regardless of their status of full-hospitalization or day-hospital regime. Cats reluctant to be trained using an underwater treadmill because of their fear of water became increasingly confident through the exposure to a minimum water level within 4 days. This was gradually increased to reach the height of the cat’s greater trochanter, which was considered by the physiotherapist to be the optimal water level.

The overall rehabilitation duration was personalized to each cat. The length of treatment was established by the physiotherapist according to the extent to which the patient’s neurological condition improved, referred to as the acquisition of involuntary ambulatory status or as the absence of any improvements seen over 6 consecutive weeks.

For each cat enrolled in the study, the specific parameters were evaluated at the time of admission, and for group 1, they were also evaluated at day 15 and, if still included in the study, at days 45 (±5) and 90 (±5). The numerical continuous variables included age, body condition score (BCS), time between onset of signs and the start of rehabilitation, and duration of physical therapy, whereas the categorical variables included breed, sex, neurological presentation (using the Olby scoring system) [13], type of lesion, voluntary micturition, presence of surgery, hospitalization during the treatment, and presence of DPP. Lesions were categorized as intervertebral disc disease, trauma, and vascular types.

Each group was subdivided according to the presence or absence of DPP (DPP group and NoDPP group, respectively). DPP was assessed by applying heavy forceps pressure to the pelvic limb digits. The lack of a conscious response in the patient (e.g., turning in the direction of the applied stimulus or similar reactions) was interpreted as an absence of DPP. In cats without DPP, at the admission and during the whole treatment, the acquisition of a SW gait was detailed. Recently, SW was defined in dogs as an independent ambulation in a “deep pain negative”(without DPP) dog, typically characterized by lack of coordination between thoracic and pelvic limbs, difficulty turning, or going backward, intermittent falling (especially when changing directions), frequently intact toe knuckling response but without hopping, and increased spasticity [12]. In this study, in addition to the features mentioned above, feline patients were identified as spinal walkers if they could walk for an extensive period of time (>15 min) and, in the case of falling, they were able to regain a standing posture without assistance and to resume walking [5].

Statistical analysis was conducted using commercial software for scientific data analysis (Past^®^, version 3.17). A Shapiro–Wilk test was used to verify the normal data distribution, whereas a Chi-squared test was used to analyze parametric and nonparametric variables. A *p*-value < 0.05 was considered to be statically significant.

In order to identify the potential variables associated with the recovery of motor function, the following parameters were considered for each patient at 15, 45 (±5), and 90 (±5) days of follow-up: breed, sex, age, BCS, clinical presentation, type of lesion, voluntary micturition, presence of surgery, hospitalization during the treatment, time between onset of signs and the start of rehabilitation, duration of physical therapy, and maintenance of DPP.

The presence or absence of a crossed extensor reflex was evaluated in order to investigate potential factors related to the possibility of achieving SW within the NoDPP group.

Finally, in order to evaluate the potential role of an intensive rehabilitation program, the final outcome, represented by independent walking, was assessed and groups 1 and 2 were compared.

## 3. Results

**Figure 1:**
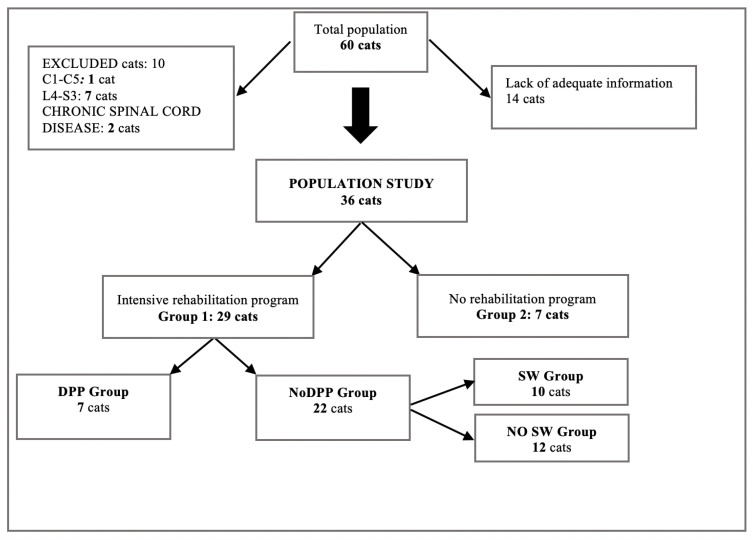
Selection of the cat population. SW, spinal walking; DPP: presence of deep pain perception; NoDPP: absence of deep pain perception.

The medical records of the 60 cats that were admitted to the physical rehabilitation center with a spinal cord injury were reviewed retrospectively. Thirty-six cats met the inclusion criteria; 29 were enrolled in the study as group 1 because they underwent an intensive physical rehabilitation protocol, and seven cats belonged to group 2 because they were managed at home without physical rehabilitation treatment (Figure 1).

Data regarding signalment, type of lesion, neurologic signs, delay in the onset of physical rehabilitation, and duration of the rehabilitation program are detailed in Table 1. 

All patients received physical therapy for a minimum of 15 days. Follow-up data obtained from recorded neurological examinations at 45 (±5) and 90 (±5) days were available for 19/29 (66%) and 8/29 (27%) cats, respectively (Table 2).

### 3.1. Group 1

#### 3.1.1. DPP Group

In the DPP group, 7/29 cats (24%) had pelvic limb DPP at the time of presentation and during the whole treatment. All of them recovered motor function before the end of the rehabilitation program. Pelvic limb DPP was significantly associated with the functional recovery of voluntary ambulatory status (*p* = 0.010) and voluntary micturition (*p* ≤ 0.001).

At the time of the first examination, 5/7 cats (71%) showed nonambulatory paraparesis, whereas the other 2/7 (29%) had ambulatory paraparesis. Micturition dysfunction affected 3/7 cats (43%) and, at the end of the treatment, two of them (67%) recovered voluntary micturition.

Spinal trauma was reported in 5/7 patients (71%), whereas the other 2/7 cats were affected by traumatic IVDH (29%). Two out of the seven cats (29%) had full-time hospitalization, whereas the others were managed on a day-hospital basis. Surgery, aimed at the stabilization of the spine after the trauma, was performed in 2/7 cases (29%) within 72 h of the trauma. None of the cats with IVDH underwent surgery because no compression of the spinal cord was detected. The time between the onset of clinical signs and the beginning of rehabilitation ranged from 3 to 271 days with a median of 44 days. The median duration of physical rehabilitation treatment was 58 days (range 19–206) (Table 3).

#### 3.1.2. NoDPP Group

In the NoDPP group, 22/29 cats (76%) showed an absence of pelvic limb DPP at the time of initial presentation and during the whole treatment. On admission, all patients were paraplegic and, at that time, 8/22 cats (36%) already showed the presence of some involuntary movements. A dysfunctional bladder was present in all cats, and partial reflex bladder emptying was reported in 3/22 (14%). At the end of the rehabilitation treatment, the acquisition of involuntary spinal ambulatory status (SW) was achieved in 10/22 cats (45%), whereas none of the patients regained a voluntary micturition.

Sixteen of the 22 cats (73%) were affected by spinal trauma (vertebral fracture/luxation), 3/22 cats (14%) had an acute traumatic IVDH, and 3/22 (14%) had a contusive/hemorrhagic injury of the spinal cord. Fifteen out of the 22 patients (68%) were hospitalized, and the others were managed in a day-hospital regime. Surgery, aimed at the stabilization of the spine, was performed in 8/22 cats (36%) within 48 h of the trauma. None of the cats with IVDH underwent surgery because the spinal cord did not appear compressed. The delay in the onset of physical therapy ranged from 5 to 900 days, and the median value was 20 days. It did not influence the outcome (*p* = 0.892). The median duration of the rehabilitation program was 46 days (range 15–302 days).

Clinical and follow-up data of the NoDPP group, expressed according to the acquisition/non-acquisition of SW, are detailed in Table 4 and Table 5, respectively. At the end of the treatment, of the 22 cats in the NoDPP group, 10 cats (45%) developed involuntary spinal ambulatory status (SW) (Table 4 and Table 5).

Involuntary spinal ambulatory status was achieved in 3/22 cats at 15 (±5) days follow-up, in 4/22 cats at the 45 (±5) day follow-up, and in 3/22 cats at the end of the rehabilitation period (>90 days) (Table 2).

On admission, the crossed extensor reflex was observed in eight cats (80%) and was significantly associated with the acquisition of SW (*p* < 0.010). 

Improvement in underwater treadmill performance, recorded in the first follow-up as an increase in the strength and start of the limb movements, was found to be significantly associated with a higher possibility of achieving SW at the end of the treatment (*p* = 0.040).

There was no significant association between the duration of the rehabilitation program and the acquisition of SW.

Breed, sex, age, BCS, type of lesion, full-time hospitalization, surgical treatment, and the presence of voluntary micturition at the time of presentation were not found to be correlated with the acquisition of SW.

### 3.2. Group 2

Group 2 included seven cats. At admission, 3/7 cats (43%) had pelvic limb DPP, whereas an absence of pelvic limb DPP was observed in 4/7 patients (57%). At the time of presentation, 6/7 patients (86%) were paraplegic, whereas the other one (14%) showed nonambulatory paraparesis. A dysfunctional bladder was reported in 6/7 cats (86%). None of the cats regained voluntary micturition or ambulatory condition at the time of the interview with the owners (>90 days from the first examination), regardless of the presence or absence of DPP at admission. Four out of seven cats (57%) were affected by spinal trauma, whereas IVDH and contusive/hemorrhagic injury were reported in 1/7 (14%) and 2/7 (29%) cats, respectively.

When comparing groups 1 and 2, the accomplishment of an intensive physical rehabilitation protocol was significantly associated with a higher possibility of regaining functional ambulatory status (*p* = 0.010). There was no significant different in the recovery of voluntary micturition between the groups. 

## 4. Discussion

Rehabilitation treatment is increasingly being considered as a crucial component of the treatment of dogs with acute onset of TL spinal cord injury and is nowadays increasingly performed in cats as well. 

To the best of the authors’ knowledge, this is the first study describing the effect of rehabilitation treatment in cats suffering from naturally occurring, acute onset of TL spinal cord injury.

In accordance with the findings reported for dogs with naturally occurring, acute onset of TL disease, our results found a positive association between the maintenance of pelvic limb DPP and the functional recovery of voluntary ambulatory status and micturition in group 1 [14,15]. The presence of DPP in pelvic limbs can be considered a clinical indicator of the residual integrity of the spinal cord and, according to the feline literature, its presence can be associated with a better outcome than its absence [16]. The absence of DPP associated with vertebral fracture/luxation is a poor prognostic indicator [17]. Surgery was performed in a small percentage of the cats with traumatic injuries and did not seem to influence the recovery. Some studies have shown that surgery can greatly influence the outcome in dogs, although a recent study on paraplegic TL dogs with absent DPP due to IVDH did not identify prognostic factors for any of the investigated variables, including early surgical treatment and severity of compression [18,19,20,21].

Experimental studies have shown that for incomplete spinal cord injuries, a positive outcome is associated with training regimens involving partial weight-bearing activity started within a critical period of one to two weeks after the injury and maintained for at least eight weeks [9]. Due to the retrospective nature of this study, there was a wider range of time in which the cat population started physical rehabilitation after injury, and the time between the onset of neurological signs and the beginning of the physical rehabilitation did not influence the outcome. It may represent a finding of great importance, because physical rehabilitation can be worthwhile and recommendable even in cats with initial acute onset of neurological signs but which are chronically paralyzed. However, this observation should be considered cautiously due to the limited number of patients.

None of the cats with DPP in group 2 regained the ability to walk, and, when comparing the cats of both groups, those that underwent an intensive physical rehabilitation treatment had a higher possibility of regaining functional ambulatory status compared with those that did not. We can speculate that the intensive physical rehabilitation in group 1 had a beneficial role, but the lack of a randomized inclusion of the cats in the groups and the small sample in group 2 could have led to bias, and this in turn may have influenced the results.

The presence of pelvic limb DPP was significantly associated with the functional recovery of voluntary micturition; however, physical rehabilitation did not seem to influence the recovery of spontaneous urination. Unfortunately, the limited and unequal number of cases in group 2 prevented us from drawing consistent conclusions.

At the end of the physical rehabilitation treatment, 10 cats (45%) without DPP in group 1 developed SW. Despite the popular opinion that a poor prognosis is associated with the absence of DPP, many studies have reported the possibility of achieving a spinal involuntary reflex gait, and some authors have also described this type of locomotion in dogs with naturally occurring, acute onset of TL spinal cord disease [5,13]. SW is commonly considered a reflex stepping gate, generated by the central pattern generator, independent of supraspinal or sensory input. However, gait generation is a complex process and different mechanisms may influence the recovery of motor function below the level of severe injury [12]. In fact, in naturally occurring spinal cord injury, development of ambulation in patients without DPP may reflect a reorganized central pattern generator in complex coordination with multiple other factors, such as spared supraspinal influence, a certain threshold of motor neuron pool excitability, peripheral sensory input, and specific locomotor training [12]. To the best of the authors’ knowledge, SW has been described in cats only in experimental contexts, and our data showed the acquisition of SW in a relevant percentage of the study population under natural conditions. Noticeably, all of these cats belonged to the group that underwent intensive physical rehabilitation.

The crossed extensor reflex was associated with the acquisition of SW, and relevant improvement in underwater treadmill performance during the first 15 days was found to be associated with a higher possibility of achieving SW at the end of the treatment. Such information might be helpful for physiotherapists and neurologists in the early identification of potential candidates for developing SW. Prospective studies are necessary to further investigate and confirm these preliminary observations. 

Most of the cats presented with dramatic ambulatory deficits, and no association between clinical presentation and final outcome was found. We cautiously hypothesize that intensive physical rehabilitation could represent an effective tool in improving the outcome, independently from the severity of neurological signs. Physical rehabilitation could play an important role in supporting the restoration of motor function, modeling and promoting the so-called “plasticity” of the spinal cord [22]. In an experimental scenario, several studies with a control group of cats not undergoing treadmill training following injury have demonstrated how this exercise produces better recovery in terms of maximum speed and number of steps taken on the treadmill [7,8], greater articular excursion, and a more symmetrical gait [8,23].

In accordance with the literature, the most frequent spinal disorder reported in our population was a traumatic external injury. Disc disease, one of the most represented causes of acute spinal cord injury (SCI) in dogs, is much less common in cats [24,25], possibly due to the minor degree of feline disc degeneration [26].

In this study, 42% of the cats that were examined by the physiotherapist did not receive intensive physical therapy, because of their owner’s refusal. This may be because they anticipated difficulties in managing feline patients, as they may not be as cooperative as dogs, especially in hydrotherapy sessions [27]. All cats underwent physical rehabilitation smoothly because different protocols were established by the physiotherapist according to the cat’s behavior. Only docile cats were immediately submitted to aggressive physical rehabilitation, including hydrotherapy, whereas for more frightened or aggressive cats, a much slower progressive treatment regimen was provided. As mentioned above, progressive exposure to water made anxious cats increasingly confident within a few days. In our study, full-time hospitalization was not associated with the better recovery of ambulatory status, leading to the conclusion that the rehabilitation protocol can also be achieved successfully in a day-hospital regimen, helping the owner in terms of cost abatement and reducing the stress for the cats.

The present study has several limitations, and caution must be taken in the interpretation of the results, since they were obtained from a small number of animals in a nonrandomized study, possibly not being a true reflection of the reliability of the data. The retrospective nature of this study did not allow us to achieve a homogeneous sample in terms of group distribution and timing in the start of physical rehabilitation, and this could have possibly produced biased data. Other limitations are due to the nonstandardized therapeutic protocol (i.e., personalization of the physical rehabilitation program), the smaller size of the control group (group 2), and the absence of an important factor affecting the outcome, namely, the implementation of specific treatments (e.g., surgery). Finally, phone calls (instead of clinical assessment) as the single evaluating means for the group that did not undertake physiotherapy may be unreliable, as subtle ambulatory status and evaluation of return of DPP are difficult for owners to assess.

Nevertheless, the authors are confident that these preliminary results may represent an interesting starting point for randomized, blinded, prospective clinical trials enrolling a large number of cats.

## 5. Conclusions

As previously described in dogs, cats with severe acute onset of TL spinal cord injury may also benefit from intensive physical rehabilitation. The present study demonstrated that, in the case of the loss of DPP, the acquisition of SW is possible in a relevant percentage of cases with naturally occurring, acute onset of TL spinal cord injury. Nevertheless, owners should be informed of the high possibility of a persistent dysfunctional bladder and the necessity of lifelong manual expression. This study provides some preliminary suggestions for further prospective investigation into the use of prognostic indicators, such as the crossed extensor reflex and early movements using an underwater treadmill, to improve the prediction of the outcome of severe TL conditions.

## Figures and Tables

**Table 1:** Data regarding signalment, type of lesion, treatment, full-time hospitalization, delay in the onset of physical rehabilitation, and duration of the rehabilitation program (group 1).

Cats		29
Breed	European shorthair	*n* = 25 (86%)
	Other	*n* = 4 (14%)
Age	m 24 months (range 1–168)	
Sex	Male	*n* = 10 (83%); C, *n* = 2 (17%)
	Female	*n* = 12 (71%); N, *n* = 5 (29%)
BCS	m = 5 (range 4–8)	
Type of lesion	Spinal vertebral trauma	*n* = 21 (73%)
	IVDH	*n* = 5 (17%)
	Contusive/hemorrhagic SC injury	*n* = 3 (10%)
Cats with full-time hospitalization		*n* = 17 (59%)
Cats undergoing spinal surgery		*n* = 3 (34%)
Delay in onset of physical rehabilitation *	m = 32 days (range 3–900 days)	
Duration of rehabilitation program	m = 47 days (range 15–302)	

* Days between the onset of early signs and the beginning of physical therapy. m, median value; C, castrated; N, neutered; BCS, body condition score; IVDH, intervertebral disk herniation; SC, spinal cord.

**Table 2:** Follow-up at 15, 45, and 90 days and at the end of treatment in the DPP group (deep pain perception present) and NoDPP group (deep pain perception absent) (group 1).

		Cats Under Intensive Physiotherapy Treatment	Cats that Stopped the Treatment Because they Recovered Voluntary Motor Function (DPP Group) or Acquired SW (NoDPP Group)	Cats that Stopped Rehabilitation Program Based on the Owner’s Decision or the Absence of Improvement
15 Days	DPP group	7/7	0/7	0/7
	NoDPP group	22/22	0/22	0/22
45 Days	DPP group	6/7	1/7	0/7
	NoDPP group	13/22	3/22	6/22
90 Days	DPP group	3/7	3/7	0/7
	NoDPP group	5/22	4/22	4/22
End of treatment	Total DPP group		7/7	0/7
	Total NoDPP group		10/22	12/22

**Table 3:** Summary of clinical data of the DPP group of cats (group 1).

		FR Cats (*n* = 7)	No FR Cats (*n* = 0)
Type of lesion	Spinal trauma	*n* = 5	-
	IVDH	*n* = 2	-
Full-time hospitalization		*n* = 2	-
Cats undergoing surgery		*n* = 2	-
Delay in onset of physical rehabilitation * (days)		m = 44 (range 3–271)	-
Duration of rehabilitation program (days)		m = 58 (range 19–206)	-

* Days between the onset of early signs and the beginning of physical therapy. m, median value; FR, functional recovery of voluntary ambulatory status; No FR, no functional recovery of l ambulatory status; IVDH, intervertebral disk herniation.

**Table 4:** Summary of the clinical data of the NoDPP group of cats (group 1).

		SW Cats (*n* = 10)	No SW Cats (*n* = 12)	*p*-Value
Type of lesion	Spinal trauma	*n* = 6	*n* = 10	
	IVDH	*n* = 2	*n* = 1	0.254
	Contusive/hemorrhagic injury	*n* = 2	*n* = 1	
Full-time hospitalization		*n* = 6	*n* = 9	0.451
Cats undergoing surgery		*n* = 4	*n* = 4	0.746
Delay in onset of physical rehabilitation * (days)		m = 26 (range 7–900)	m = 19 (range 5–730)	0.892
Duration of rehabilitation program (days)		m = 52 (range 18–302)	m = 46 (range 15–204)	0.555
Crossed extensor reflex at admission		*n* = 8	*n* = 6	0.008 **

* Days between the onset of early signs and the beginning of physical therapy. m, median value; SW, spinal walking; No SW, no spinal walking; IVDH, intervertebral disk herniation. ** Significant value *p* < 0.05.

**Table 5:** Follow-up at 15, 45, and 90 days after the initiation of the rehabilitation program in the NoDPP group (group 1). The number of cats that showed improvements in relation to the parameters evaluated is reported for each follow-up. * Significant value *p* < 0.05.

	*p*-Value (15 Days)	15 Days 45 Days 90 Days					
		SW	No SW	SW	No SW	SW	No SW
Cats		3/22	19/22	4/13	8/13	3/5	2/5
Muscle Tone	0.351	10/22	10/22	7/13	6/13	3/5	2/5
First limb movements	0.175	10/22	10/22	7/13	6/13	3/5	2/5
Assisted walking/underwater treadmill	0.043 *	10/22	8/22	7/13	6/13	3/5	2/5
Standing	0.639	4/22	6/22	7/13	4/5	3/5	2/5
Voluntary micturition	0.650	0/22	0/22	0/13	0/13	0/5	0/5

## Data Availability

The data presented in this study are available on reasonable request from the corresponding author.
